# Optoelectronic Properties of X-Doped (X = O, S, Te) Photovoltaic CSe with Puckered Structure

**DOI:** 10.3390/ma11030431

**Published:** 2018-03-16

**Authors:** Qiang Zhang, Tianyuan Xin, Xiaoke Lu, Yuexia Wang

**Affiliations:** Applied Ion Beam Physics Laboratory, Institute of Modern Physics, Fudan University, Shanghai 200433, China; qiangzhang16@fudan.edu.cn (Q.Z.); tyxin16@fudan.edu.cn (T.X.); xiaokelu15@fudan.edu.cn (X.L.)

**Keywords:** first principles, doping, electronic properties, optical properties

## Abstract

We exploited novel two-dimensional (2D) carbon selenide (CSe) with a structure analogous to phosphorene, and probed its electronics and optoelectronics. Calculating phonon spectra using the density functional perturbation theory (DFPT) method indicated that 2D CSe possesses dynamic stability, which made it possible to tune and equip CSe with outstanding properties by way of X-doping (X = O, S, Te), i.e., X substituting Se atoms. Then systematic investigation on the structural, electronic, and optical properties of pristine and X-doped monolayer CSe was carried out using the density functional theory (DFT) method. It was found that the bonding feature of C-X is intimately associated with the electronegativity and radius of the doping atoms, which leads to diverse electronic and optical properties for doping different group VI elements. All the systems possess direct gaps, except for O-doping. Substituting O for Se atoms in monolayer CSe brings about a transition from a direct Γ-Γ band gap to an indirect Γ-Y band gap. Moreover, the value of the band gap decreases with increased doping concentration and radius of doping atoms. A red shift in absorption spectra occurs toward the visible range of radiation after doping, and the red-shift phenomenon becomes more obvious with increased radius and concentration of doping atoms. The results can be useful for filtering doping atoms according to their radius or electronegativity in order to tailor optical spectra efficiently.

## 1. Introduction

Low-dimensional nanostructures, particularly monolayer honeycomb structures, attract much attention owing to their unique structure and exceptional properties, which differ from those of their bulk counterparts. Among the honeycomb structures, graphene is the most popularly investigated monolayer [1]. However, the major disadvantage of graphene is that it does not possess a nonzero band gap, and hence it is difficult to apply graphene in semiconductor devices [2,3,4,5,6]. Present studies concerned with two-dimensional (2D) materials are mainly focused on transition metal dichalcogenides (TMDs) [7,8,9,10] and single atom-thin sheets formed by group V atoms [11,12] to design novel nanoelectronic devices, such as field effect transistors and solar cells. Although these 2D materials have demonstrated many promising electronic and optoelectronic properties compared with bulk materials, their apparent disadvantages still limit their application. For instance, MoS_2_, a monolayer TMD, possesses a direct band gap [7] and self-healing properties in air [13], but its less dispersive band edges, stemming from relative localization of d electrons, which is the inherent character of transition metals [14], result in relatively heavy carrier effective mass [15,16] and thus somewhat bad mobility, disadvantaging high-performance applications [17,18]. Single atom-thin sheets constituting group V elements such as phosphorene [19], arsenene [20], antimonene [21], and bismuthene [22,23] exhibit superior performance, including high mobility [24] and excellent flexibility of adjusting properties [25,26], while their poor stability leads to rapid degradation when they are exposed to air [27,28]. Although much effort has been devoted to improving the properties of these 2D materials [29,30], there is an urgent need to discover or design novel 2D materials to overcome the above-mentioned problems. 

Group IV–VI monolayers, an emerging system of 2D families, including isoelectronic and binary counterparts of group V, have motivated extensive studies on their large-scale application in photovoltaics and thermoelectrics on account of their merits, such as abundance in the earth, environmental compatibility, lower toxicity, and chemical stability [31,32,33,34,35,36]. Recently, puckered (hinge-like) monolayers such as GeS, SiS, SnS, and SnSe have been the focus of scientific research [37,38,39,40,41], since they have a phosphorene-analogous structure. Unfortunately, it is well known that they are indirect-gap semiconductors and undesirable for practical applications. Consequently, searching for new 2D materials with promising electronic and optical properties is necessary for developing next-generation optoelectronic devices. Kamal et al. [42] systematically investigated the geometric, energetic, and electronic properties of group IV–VI binary monolayers (XY) using density functional theory (DFT). The calculations of the geometric and electronic band structure indicated that puckered CSe is a direct band gap semiconductor with a gap of 0.905 eV, which opens up the possibility of exploring their application in optoelectronic devices. C and Se atoms present a puckered surface with *sp*^3^ hybridization. Each C atom forms three covalent bonds with Se, and vice versa. Interestingly, the band structure is quite similar to that of phosphorene, implying that the electronic properties should also be similar. Herein, CSe can be considered as an alternative to 2D phosphorene. The similarity between CSe and phosphorene can be understood by the fact that the electronegativity is almost identical to C and Se. In the past few years, there has been increased attention on monolayer CSe with puckered structure [43]. Nevertheless, a comprehensive study of the optical and electric properties of puckered monolayer CSe is still missing. Furthermore, there is a desire to study the effect of X-doping (X = O, S, Te) behavior on monolayer CSe in order to tune and optimize its electric and optical properties for practical applications.

Phosphorene has recently attracted significant interest for applications in electronics and optoelectronics [11,12,24,44,45], and so, inspired by this kind of research, we replaced phosphorus atoms with C and Se atoms but preserved the number of valence electrons involved in each bond, in order to exploit advanced materials in electronics and optoelectronics. The calculation concerning phonon spectrums indeed confirmed the dynamic stability of CSe monolayer. Furthermore, monolayer CSe with puckered structure was verified to be a direct semiconductor with a band gap of 0.9 eV. Also, the geometric, electronic, and optical properties of primitive CSe monolayer were systematically characterized based on comprehensive first-principles calculations. In addition, the effect of X (X = O, S, Te) substituting Se atoms (named X-doing) was further investigated, particularly the optoelectronic properties. Our results show that X-doping (X = O, S, Te) is an efficient method to tune the band gap and optical properties of CSe monolayer and tailor its photovoltaic properties. By providing a deeper understanding of the novel properties of doping CSe monolayer, this work will pave the way toward rationally controlling the electronic and optical properties of monolayer CSe, thus opening up opportunities for a host of high-performance optoelectronic devices.

## 2. Computational Models and Method

**Figure 1:**
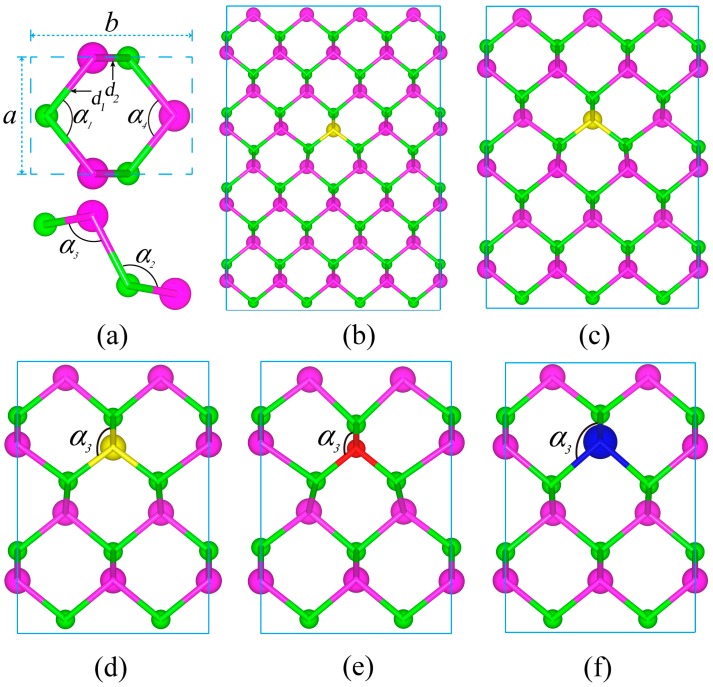
Structures of monolayer CSe with X-doping, showing two kinds of bonds with *d*_1_ and *d*_2_ lengths and the angles formed by the two bonds. (**a**) pristine CSe; (**b**–**d**) S-doping at concentrations of 1.56%, 2.78% and 6.25%, respectively; (**e**) and (**f**) O-doping and Te-doping at a concentration of 6.25%. Purple, green, yellow, red, and blue balls are Se, C, S, O and Te atoms, respectively.

DFT calculations were performed based on projector augmented wave (PAW) pseudopotentials, using the Vienna Ab initio Simulation Package (VASP) [46,47,48]. The exchange-correlation interaction was described with the generalized gradient approximation (GGA) given by Perdew-Burke-Ernzerhof (PBE) [49]. The PBE functional has been used to optimize the structures of doped and primitive monolayer CSe and to compute the phonon dispersion spectrum of primitive monolayer CSe. After optimizing the structure, the phonon curves were computed by using density functional perturbation (DFPT) theory [50]. To overcome the problem of inaccurate band structure in the PBE functional, the hybrid Heyd-Scuseria-Ernzerhof (HSE06) method [51,52,53] was used to calculate the band structures and optical properties of all the monolayers employed here. The kinetic energy cutoff for the wave function expanded in plane waves was set at 400 eV, and a vacuum space of 18 Å between adjacent single layers was adopted to avoid interlayer interactions. The convergence criteria for total force between atoms and energy in self-consistent field (SCF) cycles were chosen to be 0.01 eV/Å and 10^−6^ eV/atom, respectively. The same valence electronic configurations as Kamal [42] for C [2*s*^2^ 2*p*^2^], O [2*s*^2^ 2*p*^4^], S [3*s*^2^ 3*p*^4^], Se [4*s*^2^ 4*p*^4^] and Te [5*s*^2^ 5*p*^4^] were chosen. In the present study, an X (X = O, S, Te) atom was used to substitute an Se atom of pure monolayer CSe in order to investigate the effect of X elements on the structures and optoelectronic properties of monolayer CSe. The supercells of the puckered structure were chosen to be 2 × 2 × 1, 3 × 3 × 1 and 4 × 4 × 1, which were dependent on the S-doping concentration in primitive cells. We monitored the electronic structure of S-doping at the given concentrations 1.56%, 2.78% and 6.25%, respectively. The doping was realized by means of substituting S for Se atoms in the aforementioned supercells, as shown in Figure 1b–d. The 2 × 2 × 1 supercell was also chosen to investigate the effect of the radius of doping atoms on the structure and optoelectronic properties, where the concentrations for all X-doping (X = O, S, Te) cases were fixed at 6.25%. Monkhorst-Pack k-point sampling was set to be 41 × 31 × 1, 21 × 15 × 1, 15 × 11 × 1 and 11 × 9 × 1 for the primitive cell and X-doped supercells, respectively. Optical calculations [54] were done using the modified tetrahedron method with a grid containing more k-points in the irreducible Brillouin zone (BZ), where there were not only occupied states but also ample unoccupied states. It was verified that in the calculation of properties, HSE06 relaxation was slightly different from PBE relaxation [55]. So, all the structures were fully relaxed first using the PBE functional. The subsequent calculations were based on the PBE-relaxed structures. 

## 3. Results and Discussion

### 3.1. Geometric Structures

Puckered structure has been observed to be stable in the form of monolayer, such as phosphorene [44]. Very recently, it was reported that puckered structure with the space group *Pmn*2_1_ is the most stable configuration among the structural motifs of CSe monolayer [42]. As shown in Figure 1a, the hexagonal monolayer structure of CSe is similar to phosphorene and arsenene [44,56], where its primitive cell contains two C atoms and two Se atoms, constructing two kinds of C–Se bonds with *d*_1_ and *d*_2_ lengths. Four kinds of angles unfold in three-dimensional space, as shown in the top view and side view of the primitive cell, which correspond to the upper and bottom panels of Figure 1a, respectively. Structure optimization of puckered monolayer CSe was performed first, and then studies of electronic and optical properties were carried out. The well-optimized lattice constants were a = 3.032 Å and b = 4.301 Å, which are fairly consistent with previous theoretical results (a = 3.034 Å and b = 4.299 Å [42]). The bond lengths shown in Table 1 and bond angles (*α*_1_ = *α*_4_ = 101.39°, *α*_2_ = 100.8°, and *α*_3_ = 112.7°) are also in accord with recent results [42], indicating that our results are trustworthy. 

**Figure 2:**
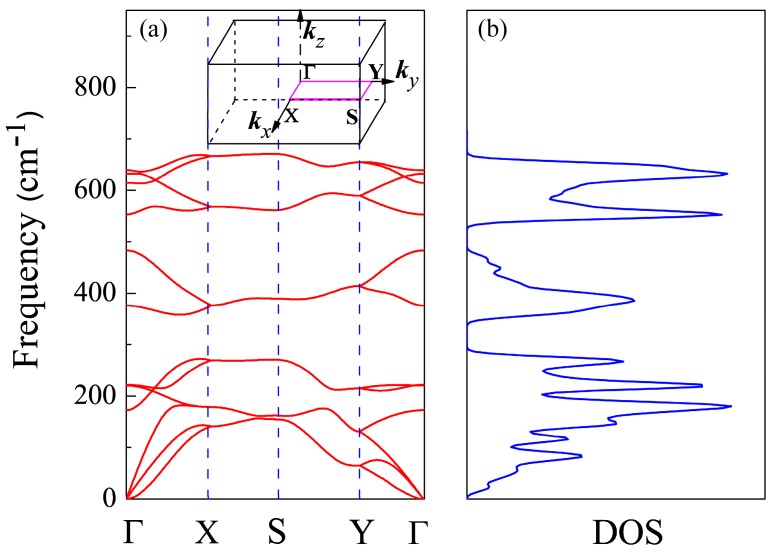
(**a**) Phonon spectrum and (**b**) density of phonon states (DOS) of CSe monolayer. The 2D Brillouin zone of CSe monolayer is shown as an insert in the phonon spectrum.

To check the stability of primitive CSe monolayer, we calculated the phonon spectrum and density of phonon states using DFPT, shown in Figure 2a,b, respectively. There are no soft vibration modes or imaginary frequencies in the phonon spectrum, confirming CSe’s resistance to spontaneous collapse [43]. The density of phonon states in Figure 2b is compatible with the phonon band dispersions in Figure 2a. The result implies that it guarantees the stability of this structure. The same methods can be found in [43,56,57,58,59].

**Figure 3:**
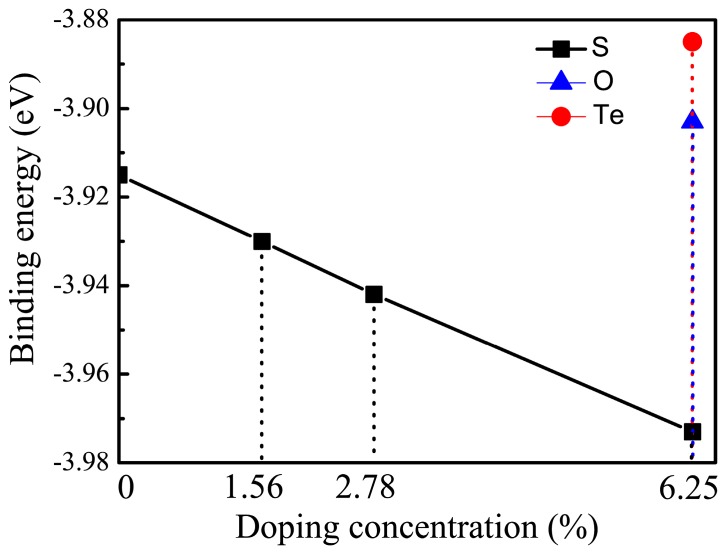
Variation of binding energy with doping elements and concentrations.

In the same group VI as Se, O, S and Te were selected as doping elements to substitute for Se. We then systematically studied the effect of the radius and concentration of doping atoms. The doping concentrations were set at 6.25%, 2.78% and 1.56%, respectively. To evaluate the plausibility of X-doping atoms in CSe monolayer, we calculated the binding energies of CSe monolayer for various doping cases. The binding energy (*E_B_*) was calculated according to the formula:

(1) $$E_{B}=(E_{\mathrm{CSe}(X)}-mE_{C(\mathrm{atom})}-nE_{\mathrm{Se}(\mathrm{atom})}-lE_{X(\mathrm{atom})})/N$$

where $E_{\mathrm{CSe}(X)}$, $E_{C(\mathrm{atom})}$, $E_{\mathrm{Se}(\mathrm{atom})}$, and $E_{X(\mathrm{atom})}$ are the energies of monolayers and C, Se and X atoms in the supercells, respectively. *m*, *n*, *l* are the numbers of C, Se and X atoms in the unit cell, and *N* is the sum of *m*, *n* and *l*. The negative value of *E_B_* indicates that the system is exothermal. The stable structures for all cases are shown in Figure 1. At the same concentration, if we artificially put two doping atoms closer in the interactive range, the binding energies of doped systems slightly increased compared with the corresponding structure shown in Figure 1. This implies that precipitation of dopants would not be favorable in energy. Figure 3 shows binding energy as a function of doping concentration concerned with the doping elements. The calculated binding energy of pristine CSe monolayer is $E_{B}$ = −3.91 eV/atom, indicating that the reaction process is exothermal and synthesizing CSe is feasible in experiments. Also, the binding energy obtained here is in good agreement with previous theoretical data ($E_{B}$ = −3.84 eV/atom [42]). It can be seen in Figure 3 that the S-doping system is energetically the most favorable, because its binding energy is lower by 0.07 and 0.088 eV/atom than that of O- and Te-doping, respectively. Hence, we focused on S-doping cases to study the doping concentration effects on optoelectronic properties. 

For the O-doping case, the C–O–C angle (*α*_3_) becomes 116.87°, much larger than the primitive cell angle (*α*_3_ = 100.8°). The O-doping monolayer is compressed along the *z* axis (perpendicular to the monolayer). For S-doping at the same concentration, the formed C–S–C angle (*α*_3_) is 104.48°, and the monolayer is slightly compressed along the *z* axis. But for Te-doping, the C–Te–C angle (*α*_3_) is 95.66°, smaller than the pristine monolayer angle, and thus the Te-doping monolayer extends along the *z* axis. Consequently, it can be seen that when the radius of doping atoms is smaller than that of Se, the monolayer will be compressed along the *z* axis, otherwise it will be extended. Compared with the three doping cases, it can be seen that severe distortion occurs in the O-doping monolayer. This may be due to Se having the most deviation of O radius and electronegativity among the group VI elements, which will lead to a short C–O bond (see Table 1). Before calculating the optoelectronic properties, we also evaluated the structure variation with S-doping concentrations. The results show that the C–S–C bond angles (*α*_3_) are 104.74°, 104.58° and 104.58°, corresponding to doping concentrations of 1.56%, 2.78% and 6.25%, respectively. The bond lengths of *d*_1_ and *d*_2_ in Table 1 are almost invariable, which is independent of the doping concentration.

### 3.2. Electronic Structure

**Figure 4:**
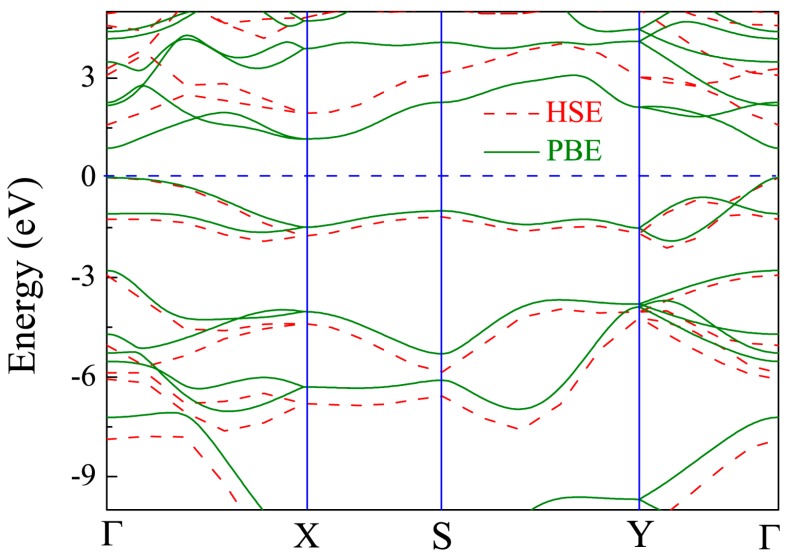
Band structure of pristine monolayer CSe. Solid line is from density functional theory (DFT)–Perdew-Burke-Ernzerhof (PBE) scheme, and dashed line from Heyd-Scuseria-Ernzerhof (HSE06). The Fermi level is set at zero.

The band structure of the primitive CSe monolayer is shown in Figure 4. The results from PBE calculations (solid line) demonstrate that the primitive CSe monolayer is a direct semiconductor at Γ (0, 0, 0), and the band gap is 0.9 eV, which is in accord with a recent theoretical prediction (0.905 eV) [42]. Noting that the band structure of CSe monolayer, including the band gap, is very close to that of phosphorene (~0.91 eV) [44,60,61], it is expected that CSe monolayer may have excellent electronic and optical properties, just as phosphorene does. Since standard PBE functional underestimates the band gap, the HSE06 method, which gives more accurate band structure [55], was adopted to repeat the calculation (see dashed line in Figure 4). The direct band gap was calculated to be 1.59 eV by HSE06, which is fairly consistent with previous theoretical results (1.58 eV) [43]. Therefore, the corrected band structure by HSE06 was used to carry out the subsequent study of optical properties.

**Figure 5:**
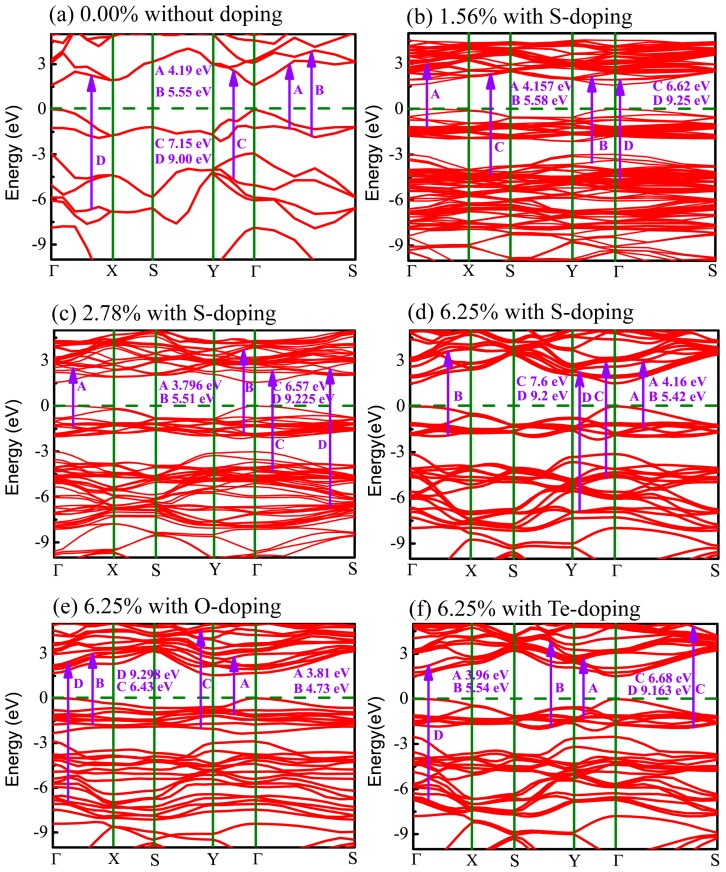
Band structure of monolayer CSe: (**a**) pristine monolayer; (**b**–**d**) S-doping at concentrations of 1.56%, 2.78%, and 6.25%, respectively; (**e**) O-doping at 6.25%; (**f**) Te-doping at 6.25%. Dashed lines specify Fermi level. Possible optical interband transitions are shown by guiding arrows.

In order to trace effects prompted by the X (X = O, S, Te) dopants, the band structures for all cases of X-doped monolayer CSe considered here are exhibited in Figure 5. It can be seen that all the monolayers are direct band-gap semiconductors with both the valence band maximum (VBM) and conduction band minimum (CBM) located at the Γ point, except the O-doped monolayer has an indirect band gap. For the O-doped monolayer CSe, VBM remains at the Γ point, while CBM transfers to the Y point. The distinguishing differences in dispersion between the O-doped monolayer and other monolayers with direct band gap, such as phosphorene, arsenene, primitive, and other doped monolayer CSe, can be attributed to the large deformation of the structure induced by O atoms, which may impact the space symmetry of monolayer CSe. Obviously, this large deformation caused only by O atoms should stem from the largest deviation of electronegativity and atomic radius of O from Se among group VI elements. In generally, the band gap of monolayer CSe becomes narrow after doping [62,63]. The value of band gap decreases with increasing doping concentrations, as can be seen in the S-doping cases in Table 2. For X-doping systems (X = O, S, Te) with 6.25% doping concentration, we also found that band gap decreases with increased doping atomic radius. The reduction in the band gap of X-doped monolayer CSe is mainly due to the downward shift of the CBM and unchanged Fermi level after doping. The details of resulting optical interband transitions shown in Figure 5 will be discussed in the next section.

It can be seen in Figure 3 that the S-doping system is energetically the most stable. Hence, we focused on S to study the doping concentration effects on optoelectronic properties. We also investigated the concentration effects of O and Te at 1.56%, 2.78% and 6.25%. The results for O and Te exhibit a similar trend to S. Moreover, S-doping sufficiently reflected the effect of doping concentration on the optoelectronic properties of monolayer CSe. Thus, we have carried out detailed discussions about the concentration effect of S.

**Figure 6:**
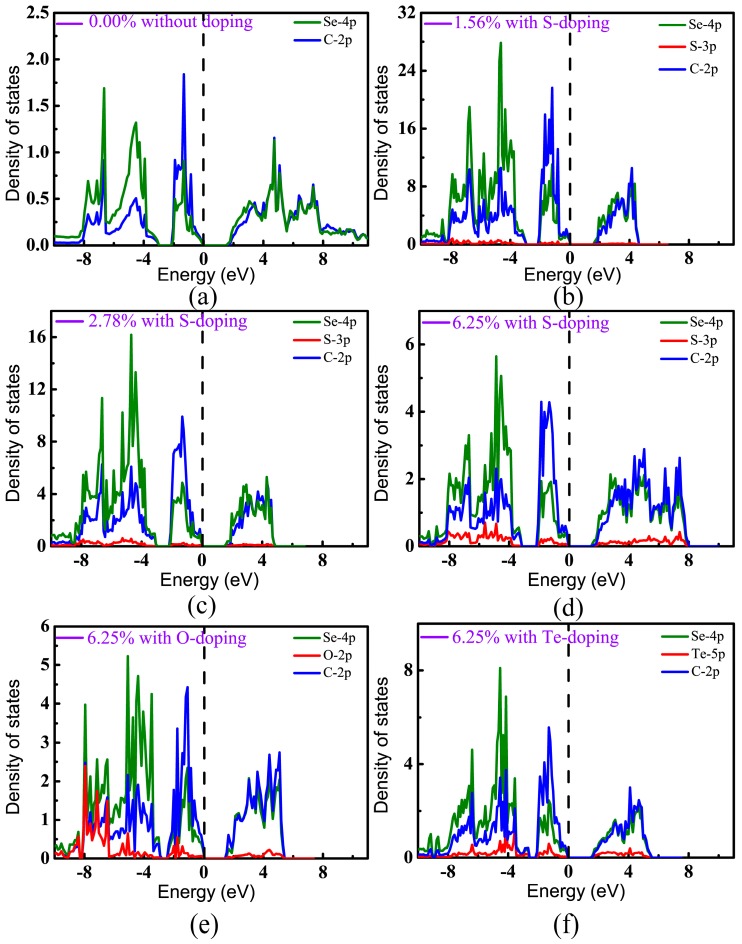
Projected densities of states (PDOS) of monolayer CSe: (**a**) pristine monolayer; (**b**–**d**) S-doping cases at concentrations of 1.56%, 2.78%, and 6.25%, respectively; (**e**) O-doping at 6.25%; (**f**) Te-doping at 6.25%. VBM is set at zero.

In order to distinguish the contributions of different orbitals to the band structures, projected densities of states (PDOS) have been calculated, as shown in Figure 6. It can be seen that the valence bands of pristine CSe were derived mainly from the C-2*p* and Se-4*p* states, which hybridize strongly with each other and form a C–Se covalent bond. As can be seen in Figure 6a–f, the valence states near the Fermi level predominantly originate from the 2*p* states of C atoms, which is in accord with a recent theoretical prediction [42]. For the conduction band, the contributions of *p* orbitals from C are nearly equivalent from Se atoms, and are relatively small from the *p* states of dopants at low doping concentration. However, with increasing doping concentration, the contributions of impurity states to valence and conduction bands become strong. Figure 6d–f show that the *p* orbitals of the doping elements mainly locate at the lower energy level of the valence band, which means that doping hardly changes the distribution of VBM from C and Se. On the other hand, doping really lowers CBM (see Table 1). So, we can understand that the band gap is decreased after doping.

To gain further insight into bonding features after doping, we carried out a Bader charge analysis to monitor charge transfer between the group IV (C) and VI (O, S, Se, Te) atoms. Bader charge was calculated, and all cases concerned with Bader charge analysis are summarized in Table 3. It clearly shows that there are always little net charges transferring between the different species in pristine CSe monolayer, indicating that weak ionic bonds are somewhat generated, which is different from the purely covalent bonding formed between the same atoms in elemental monolayers [44]. After doping, charge transfer also occurs between dopants and C atoms. O has higher electronegativity than C and Se, therefore O captures electrons from C, and C has a little net charge, although it snatches charges from its neighboring Se atoms. For S- and Te-doping, C always obtains charge from doping atoms, but gets more from Te atoms. The transfer amount between the dopants and C is consistent with the order of the electronegativity of the doping atoms: the order of electronegativity in the Pauling scale is $\xi_{O}>\xi_{C}>\xi_{S}>\xi_{\mathrm{Se}}>\xi_{\mathrm{Te}}$ for C, O, S, Se and Te values of 2.57, 3.44, 2.56, 2.55 and 2.1 eV, respectively [42]. In any case, covalent bonding is the main feature in monolayer CSe, since charge transfer is small, as seen in Table 3. Table 1 lists the bond lengths. C–X (X = O, S) bond lengths are much shorter than C–Se bond lengths, and C–Te bonds present the opposite situation, reflecting covalent features in the following order at the same doping concentration: C–O > C–S > C–Se > C–Te. It is worth noting that the electronegativity of O and Te severely deviates from that of Se, while S is comparable to Se. This may explain why the binding energy is lowest for S-doping. 

### 3.3. Optical Properties

The optical properties of materials rely substantially on the electrical properties. The desirable optical properties of monolayer CSe are expected to be obtained by effectively tuning its electrical properties with the aid of X-doping. It is well known that the dielectric function is derived from the interaction between photons and electrons, and accurately describes the optical properties of the material by means of its linear response to electromagnetic radiation on the surface [64]. The frequency-dependent dielectric function is divided into real and imaginary parts: $\varepsilon (\omega )=\varepsilon_{1}(\omega )+i\varepsilon_{2}(\omega )$. The imaginary part, $\varepsilon_{2}(\omega )$, is calculated directly using the electronic properties through the joint density of states and elements of the optical matrix between occupied and unoccupied electronic states according to the following equation [65]:

(2) $$\varepsilon_{2}=\frac{2\pi e^{2}}{\Omega \varepsilon_{0}}\sum_{k,v,c}|\langle \varphi_{k}^{c}|u\cdot r|\varphi_{k}^{v}\rangle \delta \left(E_{k}^{c}-E_{k}^{v}-\hbar \omega \right)$$

where Ω is the volume of the elementary cell and ω is the light frequency. $\varphi_{k}^{c}$ and $\varphi_{k}^{v}$ are the conduction band and valence band wave functions at k point, respectively. **u** is the vector defining the polarization of the electric field of the incident light, which is averaged over all spatial directions in the polycrystalline case. The other signs have their common meanings. The real part, $\varepsilon_{1}(\omega )$, is decided by the imaginary part $\varepsilon_{2}(\omega )$ of dielectric function using the Kramers–Kronig relations [66]:

(3) $$\varepsilon_{1}(\omega )=1+(\frac{2}{\pi })p\int_{0}^{\infty }d\omega^{\prime }\frac{{\left(\omega^{\prime }\right)}^{2}\varepsilon_{2}(\omega )}{{\left(\omega^{\prime }\right)}^{2}-{\left(\omega \right)}^{2}}$$

where *p* denotes the integral principal value. 

**Figure 7:**
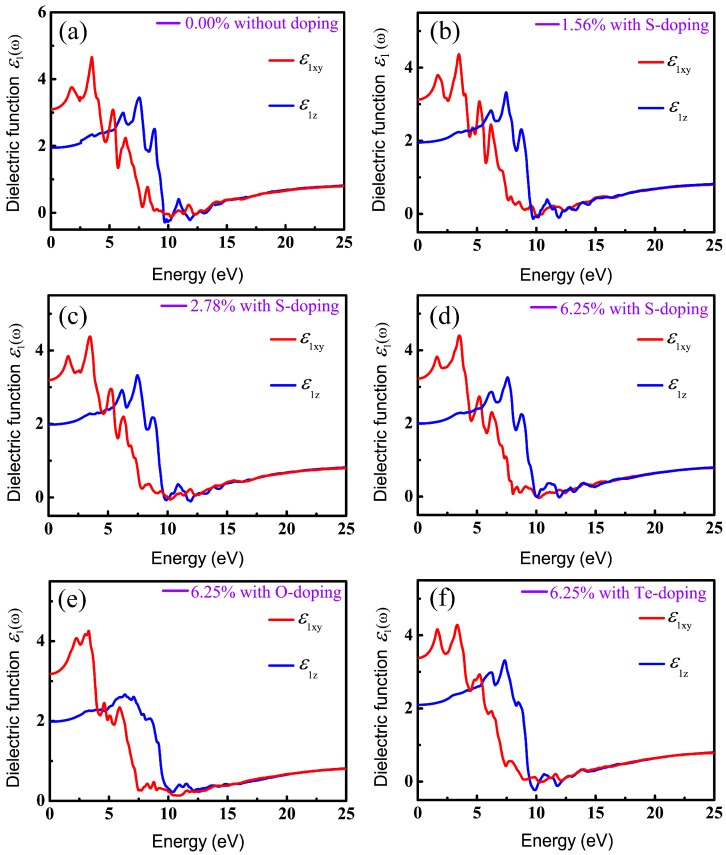
Real parts of dielectric function $\varepsilon_{1}(\omega )$ in *xy* plane and along *z* direction, versus photon energy: (**a**) pristine monolayer CSe; (**b**–**d**) S-doping at concentrations of 1.56%, 2.78% and 6.25%, respectively; (**e**) O-doping at 6.25%; (**f**) Te-doping at 6.25%.

**Figure 8:**
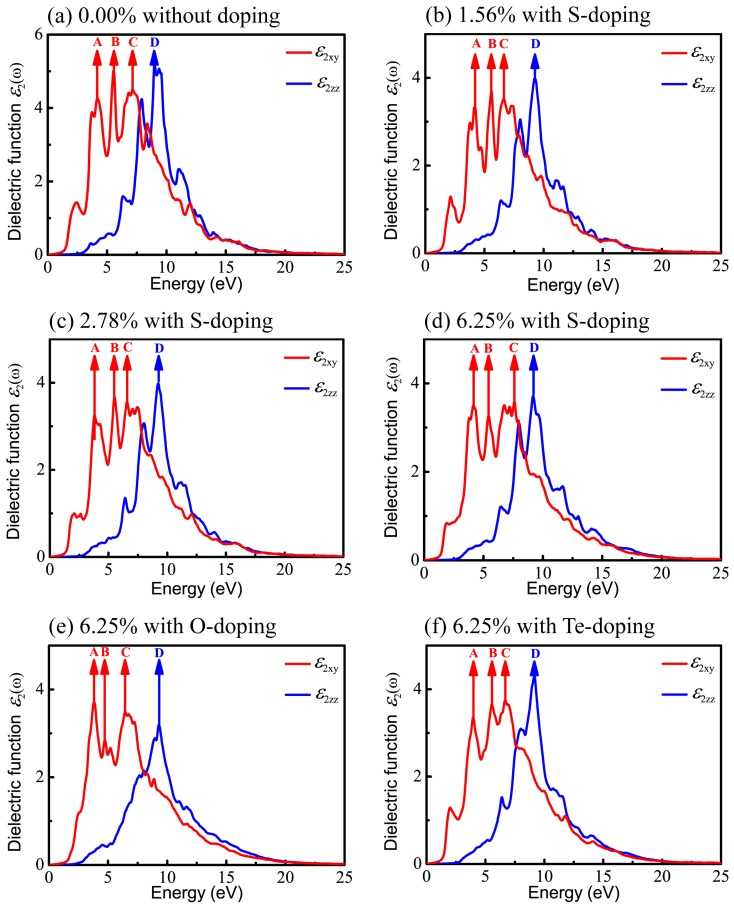
Imaginary parts of dielectric function $\varepsilon_{2}(\omega )$ in *xy* plane and along *z* direction, versus photon energy: (**a**) pristine monolayer CSe; (**b**–**d**) S-doping at concentrations of 1.56%, 2.78%, and 6.25%, respectively; (**e**) O-doping at 6.25%; (**f**) Te-doping at 6.25%.

It is known that DFT within PBE generally underestimates the band gap of semiconductors and then results in inaccurate optical calculations; however, HSE06 used here can give reasonable predictions about optical properties compared to experimental results [62,63,67]. Therefore, all optical properties stemming from the consequences of the imaginary part of the dielectric functions are based on HSE06. Also, in order to have a comprehensive understanding of the relationship between optical properties and X-doping, we systematically calculated the imaginary part of the dielectric function under various doping conditions. The real ($\varepsilon_{1}(\omega )$) and imaginary ($\varepsilon_{2}(\omega )$) parts of the complex dielectric function in *xy* plane (parallel to the monolayer) and along the *z* axis for six different X-doping cases are presented in Figure 7 and Figure 8. 

The static dielectric constant, $\varepsilon_{1}(0)$, an important parameter, is derived from the real part $\varepsilon_{1}(\omega )$ of the dielectric function at zero frequency. More importantly, band gap can be evaluated by the static dielectric constant. Table 4 lists the static dielectric constant $\varepsilon_{1}(0)$ both parallel and perpendicular to the *z* axis, with and without X-doping systems, calculated according to Equation (3). Table 2 and Table 4 indicate that the larger $\varepsilon_{1}(0)$ is, the narrower the energy gap is. This is in good agreement with the Penn model (Equation (4)) in [68], which is described as follows:

(4) $$\varepsilon_{1}(0)=1+{\left(\frac{h\omega_{p}}{E_{g}}\right)}^{2}$$

where $h\omega_{p}$ is the plasma energy and *E_g_* is the energy gap. In indirect semiconductors, successful transition of electrons from valence to conduction band needs weak phonon-assisted absorption, which will require materials equipped with considerable slab thickness to generate phonons. For instance, the typical absorber thickness of C–Si solar cells is ~200 μm [69]. The thickness of monolayer CSe studied here is less than 1 μm. Moreover, indirect transition between two different symmetry points of ***k*** space just slightly contributes to the absorption spectra. Therefore, we just considered the direct optical transition for it to primarily determine the optical properties of monolayer CSe. *E_g_* in Equation (4) is defined as a direct energy gap [62]. Using $\varepsilon_{1}(0)$ from Table 4, we also deduced *E_g_* according to Equation (4), and prioritized as follows: *E_g_* (0.00%) > *E_g_* (1.56%) > *E_g_* (2.78%) > *E_g_* (6.25%) for S-doping; *E_g_* (O-doping) > *E_g_* (S-doping) > *E_g_* (Te-doping) at the same doping concentration of 6.25%. The order is consistent with band structures analysis in Figure 5 and our calculation is proved to be reliable. 

It can be seen in Figure 7 and Figure 8 that the overall profiles of the primitive and doping systems are very similar, except for the starting position and peak values of optical spectra. In this paper, we only considered the case of incident radiation with linear polarization parallel and perpendicular to the *z* axis. Therefore, the real part $\varepsilon_{1}(\omega )$ can be decomposed into $\varepsilon_{1xy}(\omega )$ in the *xy* plane (electric vector E perpendicular to the *z* axis) and $\varepsilon_{1z}(\omega )$ along the *z* direction (E parallel to the *z* axis), as shown in Figure 7. We can see that the intensities of $\varepsilon_{1xy}(\omega )$ and $\varepsilon_{1z}(\omega )$ increase gradually with increasing photon energy, reach the maximum around 3.33–3.50 eV and 7.33–7.58 eV, and then drop gradually, approaching zero in the high-photon-energy region. The dielectric functions are found to be highly anisotropic in the low-energy range (<10 eV) and become isotropic in the highly energetic range, which is related to the 2D-structure of monolayer CSe. 

Figure 8 shows the imaginary part of the dielectric function, still decomposed into the vertical component $\varepsilon_{2xy}(\omega )$ (red curve) and the parallel component $\varepsilon_{2z}(\omega )$ (blue curve). The peaks in Figure 8 denoted by arrows imply the potential electronic transitions. With the benefit of the energy peaks, we can retrace the possible interband transitions in Figure 5, which correspond to the transition processes shown by arrows A, B, C, and D. The energy positions of four peaks of $\varepsilon_{2}(\omega )$ are listed in Table 4. 

For primitive monolayer CSe, three strong major peaks located at points A (4.19 eV), B (5.55 eV), and C (7.15 eV) of $\varepsilon_{2xy}(\omega )$ and one strong peak at point D (9.00 eV) of $\varepsilon_{2z}(\omega )$ are labeled in Figure 5a and Figure 8a. The first peak A may be due to the electron transitions between occupied states at about −1.32 eV and the unoccupied states at about 2.87 eV according to the band structure (labeled as A in Figure 5a). The transition between the occupied state at −1.77 eV and the unoccupied state at 3.78 eV may bring about peak B. Peak C is inferred mainly from the transition from −4.52 eV in valence band to 2.63 eV in conduction band. Moreover, peak D is mainly attributed to the transition between the electronic states at about −6.66 eV and at about 2.34 eV above the Fermi level.

Similar to the primitive monolayer, four primary peaks of the imaginary part marked by A, B, C, and D in Figure 8b–f are formed in $\varepsilon_{2}(\omega )$ for various X-doping cases, which also correspond to the possible optical interband transitions presented in electronic band structures (see Figure 5b–f). The three prominent peaks (A, B and C) of $\varepsilon_{2xy}(\omega )$ and one peak (D) of $\varepsilon_{2z}(\omega )$ formed at 1.56% S-doping concentration mainly originate from interband transitions −1.195 → 2.962 eV, −3.55 → 2.03 eV, −4.36 → −2.26 eV, and −4.73 → 4.52 eV, respectively. Figure 8c shows that, at 2.78%, the four peaks (A, B, C, D) come from interband transitions −1.3 → 2.496 eV, −1.8 → 3.71 eV, −4.3 → 2.27 eV, and −6.725 → 2.5 eV, respectively. For the 6.25% S-doping case, the transitions −1.39 → 2.77 eV, −1.9 → 3.52 eV, −4.65 → 2.95 eV, and −6.9 → 2.3 eV constitute the four prominent peaks (A, B, C, D), respectively, in Figure 8d. For O-doping at the concentration of 6.25%, the four crucial peaks (A, B, C and D) originate from interband transitions −1.09 → 2.72 eV, −1.77 → 2.96 eV, −2 → 4.43 eV, and −6.92 → 2.378 eV, respectively. For the 6.25% Te-doping case, A, B, C and D peaks rely on the interband transitions −1.3 → 2.66 eV, −1.89 → 3.75 eV, −1.89 → 4.79 eV, and −9.443 → −0.28 eV, respectively. Shown by $\varepsilon_{2xy}(\omega )$, all the A peaks lie in the ultraviolet (UV) region of the solar spectrum. This indicates that primitive CSe and all doping cases are good absorbers in the UV region, which predicts the practicality of monolayer CSe in solar cells as a window layer or top junction in tandem solar cells. We should clarify that quite a few transitions with the same energy located at the same peak position in the imaginary part of the dielectric function ($\varepsilon_{2}(\omega )$) may occur, but from the different interbands. So, the transitions, denoted in Figure 5 and Figure 8, are just part of the possible interband transitions. Considering there is limited experimental data on the dielectric spectra, we expect that the results can act as a prediction for further experiments.

Next, we discuss the effect of doping on the absorption spectra. The absorption coefficient, α(ω), can be calculated using the real and imaginary parts of the complex dielectric function as follows:

(5) $$\alpha (\omega )=\sqrt{2}\omega^{1/2}{\left(\sqrt{\varepsilon_{1}^{2}(\omega )+\varepsilon_{2}^{2}(\omega )}-\varepsilon_{1}(\omega )\right)}^{1/2}$$

**Figure 9:**
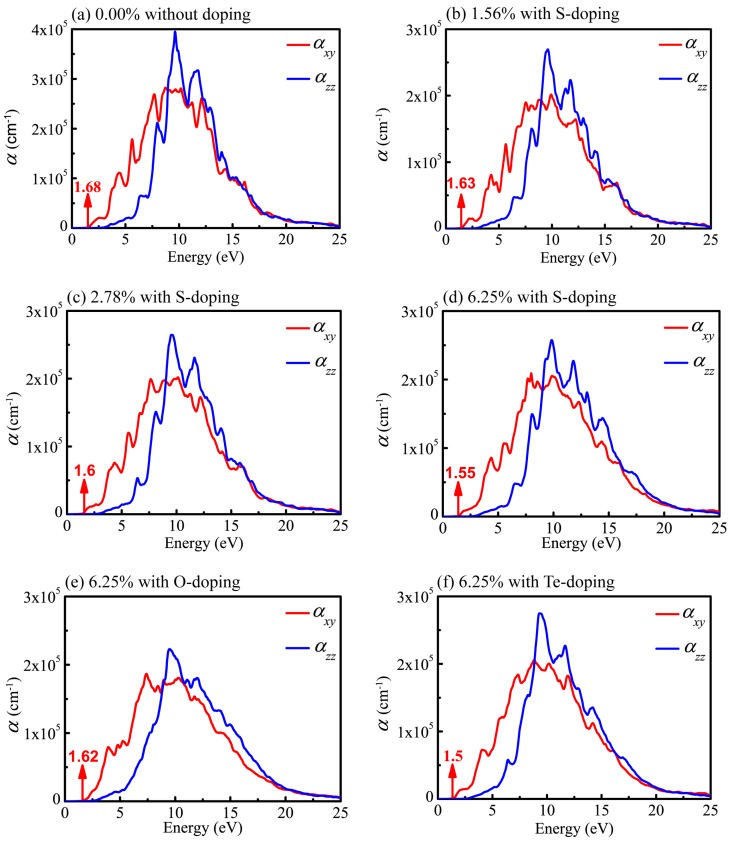
Absorption spectra of monolayer CSe in *xy* plane (red line) and along *z* axis (blue line). (**a**) pristine monolayer CSe; (**b**–**d**) S-doping at concentrations of 1.56%, 2.78% and 6.25%, respectively; (**e**) O-doping at 6.25%; (**f**) Te-doping at 6.25%.

As can be seen from the curves of the absorption coefficient in Figure 9, the values decrease rapidly in the low-energy region, which is a conspicuous feature of the semiconductor and insulator. It is clear that the absorption coefficients in the *xy* plane ($\alpha_{xy}$) and along the *z* axis ($\alpha_{zz}$) show apparent disparity. Besides, the absorption spectra (Figure 9) for the six cases considered here display profiles in the whole wavelength range nearly the same as those of the undoped system. However, the intensities of peaks are slightly weakened in the doping system. This originates from the reduction in both $\varepsilon_{1}(\omega )$ and $\varepsilon_{2}(\omega )$ after doping as a consequence, leading to the weaker intensities of absorption peaks according to Equation (5). In any case, the results indicate that the optical properties are similar in the six systems. 

As shown in Figure 9a, the absorption edge of pristine monolayer CSe is about 1.68 eV, below which almost no optical absorption takes place. It is obvious that the optical absorption edge (1.68 eV) is very close to the band gap (1.59 eV). Generally, absorption edge is positively correlated with band gap. Red shift of the adsorption edge will occur after doping. For S-doping, shown in Figure 9b–d, the absorption edges move to 1.63, 1.60 and 1.55 eV with the concentrations changing to 1.56%, 2.78%, and 6.25%, respectively. At a concentration of 6.25%, shown in Figure 9d–f, the adsorption edges are 1.62, 1.55 and 1.5 eV for O-, S- and Te-doping, respectively, indicating that red shift is more obvious with increased doping atom radius in the same group of elements. In addition, doping has little effect on the width of the absorption spectra, but evidently adjusts the intensities of absorption peaks. For S-doping cases, with increased concentration, the intensity of the absorption becomes stronger. At the same concentration of 6.25%, the intensities of absorption slightly decrease when atomic radius size moves from Te to O atoms, which is similar to the situation of LiInS_2_ and LiInSe_2_ [55]. This is likely caused by the diverse electronegativity of doping atoms. As mentioned above, the order of electronegativity for group VI elements is $\xi_{O}>\xi_{S}>\xi_{Te}$. The radius of O element is the smallest, and its electronegativity is the largest. Its strong electronic localization disadvantages electronic transitions. Therefore, the intensity of absorption decreases with decreased doping atomic radius. Since there is a lack of experimental data on the absorption spectra, an evaluation of our theoretic results is expected. Such calculations may stimulate further experimental investigations.

## 4. Conclusions

In this paper, we systematically investigated the geometric, electronic, and optical properties of group IV–VI primitive CSe monolayer as well as X-doping (X = O, S, Te) by means of first-principle calculations based on PBE-optimized crystal geometries. The optimized geometry was consistent with previous theoretical results. We found that for O and S doping, the C–X bond is always shorter than the C–Se bond. On the other hand, a longer C–Te bond will be formed with Te-doping. This is intimately associated with the bonding feature of C–X, where the C–O and C–S bonds possess stronger covalence, and C–Te has weaker covalence, compared with the C–Se bond. Because the electronegativity of O is deviates the most from Se, it induces severe deformation of monolayer CSe, therefore a transition from a direct Γ-Γ band gap to an indirect Γ-Y band gap. The nature of direct band gap of CSe remains to be described for S- and Te-doping. For S-doping, the value of direct band gap decreases with increased concentration. At a given doping concentration, band gap becomes narrow when the radius of doping atoms is increased. 

Doping expands the range of optical adsorption. The red-shift phenomenon occurs after doping, and becomes more obvious when the radius and concentration of doping atoms are increased. Moreover, we found that the optical properties can be efficiently tuned via filtering the radius or electronegativity of dopants. This will enrich the methods to tailor the electronic and optical properties of 2D materials to satisfy the demand for optoelectronics devices. We expect that the highly tunable optical properties of X-doping CSe compounds can be applied in the field of optoelectronics. 

## Figures and Tables

**Table 1:** Geometric parameters, Fermi level, and conduction band minimum (CBM) of monolayer CSe for X-doping (X = O, S and Te) with different concentrations.

Doping Atoms	Concentration	Bond Type	Bond Length		Fermi Level	CBM
X	(%)	-	(Å)		(eV)	(eV)
			*d*_1_	*d*_2_		
Pristine CSe	0.00	C–Se	1.961	2.016	0	1.59
S	1.56	C–Se	1.968	2.019	0	1.52
-	-	C–S	1.770	1.839	-	-
S	2.78	C–Se	1.969	2.020	0	1.48
-	-	C–S	1.775	1.838	-	-
S	6.25	C–Se	1.969	2.020	0	1.43
-	-	C–S	1.789	1.838	-	-
O	6.25	C–Se	1.983	2.057	0	1.54
-	-	C–O	1.662	1.490	-	-
Te	6.25	C–Se	1.957	2.004	0	1.39
-	-	C–Te	2.116	2.178	-	-

**Table 2:** Band gaps of monolayer CSe with and without doping.

Doped Atoms X	Concentration (%)	Band Gap (eV)	CBM	VBM
-	0.00	1.59	Γ	Γ
S	1.56	1.52	Γ	Γ
S	2.78	1.48	Γ	Γ
S	6.25	1.43	Γ	Γ
O	6.25	1.54	Y	Γ
Te	6.25	1.39	Γ	Γ

**Table 3:** Bader charge of X and C atoms, net charge transfer, and the difference in electronegativity ($\xi_{X}-\xi_{C}$) between X and C atoms in X-doping CSe.

Doped Atoms	Concentration	Bader Charge (e)			Charge Transfer (e)		$\xi_{X}\;-\;\xi_{C}$
X	(%)	C	Se	X	From Se to C	From X to C	(eV)
-	0.00	4.647	5.353	-	0.216	-	-
S	1.56	4.5832	5.3349	5.5876	0.2229	0.1375	0.01
S	2.78	4.5805	5.348	5.5919	0.2223	0.136	0.01
S	6.25	4.561	5.3550	5.621	0.2174	0.1263	0.01
O	6.25	4.115	5.3933	6.7682	0.1856	−0.2561	0.87
Te	6.25	4.7933	5.3471	4.8877	0.2113	0.3708	–0.47

**Table 4:** Static dielectric constant $\varepsilon_{1}(0)$ , peak values of imaginary part $\varepsilon_{2}(\omega )$ for monolayer CSe with and without doping.

Doped Atoms X	Concentration (%)	$\varepsilon_{1xy}(0)$	Peak Positions (eV)
-	0.00	$\varepsilon_{1xy}(0)=3.0973$	A = 4.19
-	-	$\varepsilon_{1z}(0)=1.9475$	B = 5.55
-	-	-	C = 7.15
-	-	-	D = 9.00
S	1.56	$\varepsilon_{1xy}(0)=3.1277$	A = 4.157
-	-	$\varepsilon_{1z}(0)=1.9551$	B = 5.58
-	-	-	C = 6.62
-	-	-	D = 9.25
S	2.78	$\varepsilon_{1xy}(0)=3.1196$	A = 3.796
-	-	$\varepsilon_{1z}(0)=1.9187$	B = 5.51
-	-	-	C = 6.57
-	-	-	D = 9.225
S	6.25	$\varepsilon_{1xy}(0)=3.2257$	A = 4.16
-	-	$\varepsilon_{1z}(0)=1.9957$	B = 5.42
-	-	-	C = 7.60
-	-	-	D = 9.20
O	6.25	$\varepsilon_{1xy}(0)=3.1767$	A = 3.81
-	-	$\varepsilon_{1z}(0)=1.9803$	B = 4.73
-	-	-	C = 6.43
-	-	-	D = 9.298
Te	6.25	$\varepsilon_{1xy}(0)=3.3783$	A = 3.96
-	-	$\varepsilon_{1z}(0)=2.098$	B = 5.54
-	-	-	C = 6.68
-	-	-	D = 9.163
